# A hyporeflective space between hyperreflective materials in pigment epithelial detachment and Bruch’s membrane in neovascular age-related macular degeneration

**DOI:** 10.1186/1471-2415-14-159

**Published:** 2014-12-16

**Authors:** Ryo Mukai, Taku Sato, Shoji Kishi

**Affiliations:** Department of Ophthalmology, Gunma University, School of Medicine, 3-39-15 Showa-machi, Maebashi, Gunma, 371-8511 Japan

**Keywords:** Age-related macular degeneration, Optical coherence tomography, Pigment epithelial detachment, Bruch’s membrane

## Abstract

**Background:**

The purpose of this study was to investigate the clinical characteristics of a hyporeflective space between hyperreflective materials in pigment epithelial detachment (PED) and Bruch’s membrane in neovascular age-related macular degeneration (AMD) using spectral-domain optical coherence tomography (SD-OCT) or swept source optical coherence tomography (SS-OCT).

**Methods:**

Among 223 patients with neovascular AMD, 227 eyes were studied retrospectively. Using SD-OCT or SS-OCT, we reviewed clinical characteristics of the space.

**Results:**

Twenty-two (10%) of the 227 eyes showed a space between hyperreflective materials in PED and Bruch’s membrane. In all spaces, fibrovascular changes of the choroidal neovascularization (CNV) membrane were seen on funduscopy, with OCT images showing the retinal pigment epithelium (RPE) above the space adhering tightly and continuously to the CNV membranes. Nineteen (86%) of the 22 eyes with this cleft also had serous retinal detachment or cystoid macular edema. Five eyes (23%) had an RPE tear during follow-up.

**Conclusions:**

A hyporeflective space between hyperreflective materials in PED and Bruch’s membrane sometimes appears in neovascular AMD. The appearance of such a space may indicate residual activities of the hyperreflective materials.

## Background

Age-related macular degeneration (AMD) is the leading cause of blindness in the elderly [1, 2]. Recent advances in both spectral-domain optical coherence tomography (SD-OCT), such as improvements in registration and averaging techniques of imaging and enhanced depth imaging [3] and swept source optical coherence tomography (SS-OCT), have enabled visualization of the structure of the sub-retinal pigment epithelium (RPE) in AMD. Coscas reported finding an elevation of the RPE and accumulation of material posterior to the RPE [4]. Block et al. described growth of subfoveal fibrosis around RPE in classic choroidal neovascularization (CNV) [5]. Polypoidal choroidal vasculopathy (PCV) is the entity that may occur as a form of type 1 CNV [6]. The prevalence of PCV in Asians and blacks appears to be higher than that observed in white patients [7, 8]. Indocyanine green angiography has been used to describe polypoidal lesions and the choroidal vascular network that lies beneath the RPE [9]. SD-OCT has demonstrated the presence of the sub-RPE double layer sign in the choroidal vascular network [10].

Spaide used the enhanced depth imaging-OCT technique to examine patients with pigment epithelial detachment (PED) and described the internal structure of PED associated with AMD [3]. Imaging results showed there was a hyperreflective line that was detected on the back surface of the RPE. In addition, this study also determined that PED appeared to be filled with a solid material in 11 of 22 eyes, while 10 of 11 eyes had tissue that seemed to be composed of layers or lamella with clefts. Khan et al. reported finding a hyporeflective space between the sub-RPE neovascular tissue and underlying choroid, which they referred to as the “triple-layer” sign, in 4 of 18 eyes with PCV [11]. Nagiel et al. demonstrated the presence of a hyporeflective space between Bruch’s membrane and the CNV complex in 6 of 8 eyes with an RPE tear, which they described as a “cleft” [12]. In a previous study, we have described conformational changes beneath the sub-RPE CNV in PED prior to developing a RPE tear [13].

**Figure 1:**
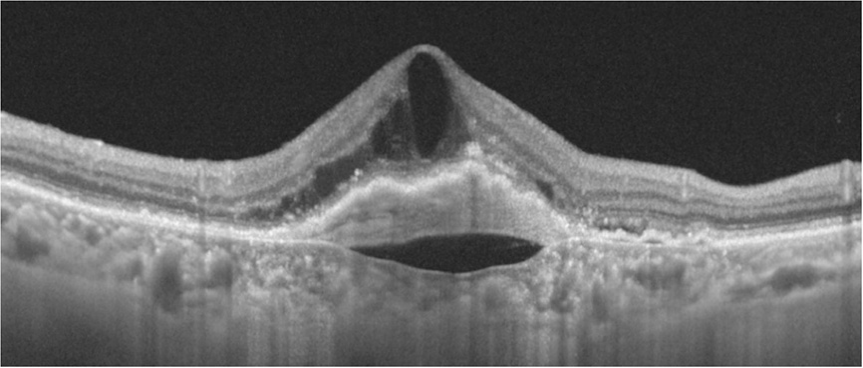
**A typical cleft in case with retinal angiomatous prolifetration.** A hyporeflective space (cleft) between Bruch’s membrane and the choroidal neovascularization complex (Case 10).

The current study used SD-OCT or SS-OCT to focus on the hyporeflective space between hyperreflective materials in PED and Bruch’s membrane, which is referred to as the “cleft” [3, 12], and clarified the clinical characteristics of this cleft in neovascular AMD (Figure 1).

## Methods

We performed a retrospective medical records review of all patients with a clinical diagnosis of neovascular AMD at the Department of Ophthalmology of Gunma University Medical Hospital from March 1, 2010 through December 31, 2011. This study was approved by the institutional review board of Gunma University Graduate School of Medicine and the research was conducted according to the provisions of the Declaration of Helsinki, 1995 (as revised in Edinburgh, 2000).

All patients were examined using a fundus ophthalmoscope, SD-OCT (Cirrus OCT; Carl Zeiss Meditec, Dublin, CA, USA, or Spectralis OCT; Heidelberg Engineering, Heidelberg, Germany), or SS-OCT (DRI OCT-1; Topcon, Tokyo, Japan). SD-OCT scans were obtained using an enhanced depth imaging technique [3]. In addition, all patients underwent vertical, horizontal, and 3-dimensional scans at the fovea, along with fluorescein and indocyanine green angiography. Subtypes of AMD were classified into four types that included: 1) typical occult CNV with no classic component; 2) typical predominantly classic CNV; 3) PCV; or 4) retinal angiomatous proliferation (RAP). We investigated the prevalence of the cleft sign among these four AMD subtypes, the maximum thickness of the cleft measured using the caliper function of the Cirrus, Spectralis, or SS-OCT instruments, the maximum thickness of the CNV membrane beyond the cleft, and the prevalence of subretinal and retinal edema associated with the cleft. We also evaluated features related to the number of patients with complete absorption of the cleft, recurrence rate of the cleft, and whether the cleft was accompanied by RPE tear. PDT with verteporfin (Visudyne; QLT, Vancouver, Canada) was administered to patients with a VA below 20/30, in accordance with the protocol of the “Treatment of Age-Related Macular Degeneration with Photodynamic Therapy Study Group” [14]. Treatment was administered using a 689-nm laser system (Carl Zeiss Meditec) that delivered 50 J/cm^2^ of energy during an 83-s exposure time. A single injection of ranibizumab (Lucentis, 0.5 mg/0.05 ml; Genentech, South San Francisco, CA, USA) was administered 2 days before application of PDT. Over the next 2 months, additional intravitreal injections of ranibizumab were administered each month. Ranibizumab monotherapy was used for patients with a VA exceeding 20/30. Three ranibizumab injections were given as a loading dose, with injections administered at approximately 4-week intervals.

When recurrent or residual exudative changes were seen on OCT images, an additional injection of ranibizumab was administered. In Case 6, the patient had undergone PDT monotherapy because of cervical arterial stenosis.

A chi-square test was used to analyze whether the prevalence of the cleft was correlated to AMD subtype. Analysis of data was performed using SPSS version 20.0 (IBM Japan, Tokyo, Japan).

## Results

**Figure 2:**
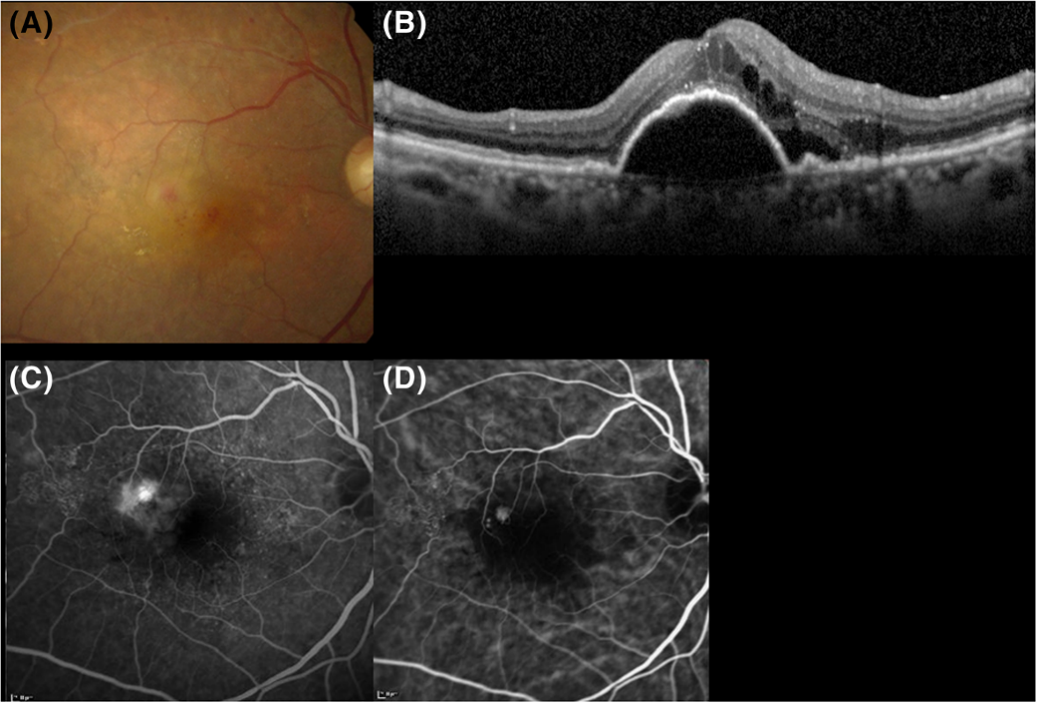
**Initial presentation of Case 10.** Retinal angiomatous proliferation at the initial visit. **A)** A fundus photograph shows an intraretinal blot hemorrhage at the fovea. **B)** Spectral-domain optical coherence tomography (vertical scan) shows subretinal fluid and cystoid macular edema with pigment epithelial detachment. **C)** Fluorescence angiography shows leakage from intraretinal neovascularization and intraretinal edema. **D)** Indocyanine green angiography shows intraretinal neovascularization accompanied by retinal-retinal anastomosis. The right eye was treated with photodynamic therapy combined with three consecutive intravitreal injections of ranibizumab.

**Figure 3:**
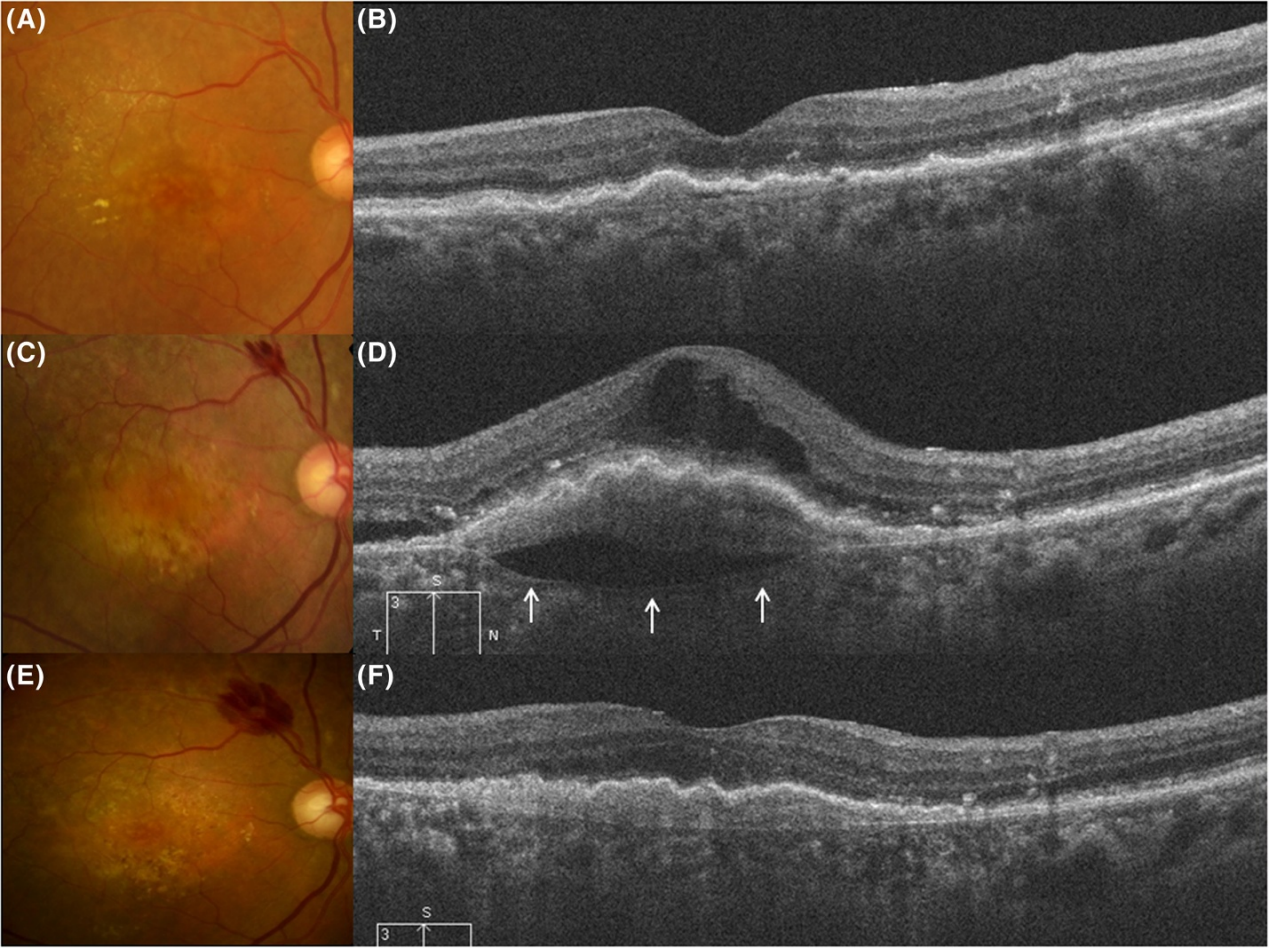
**Clinical course of the cleft in Case 10.** Clinical courses of fundus and results of spectral-domain optical coherence tomography (SD-OCT) (vertical scans) in Case 10 at 3 months after first treatment **(A,B)**, at 24 months **(C,D)** and at 25 months **(E,F)** after first treatment. **A)** A fundus photograph shows resolution of the intraretinal hemorrhage. **B)** SD-OCT shows absorption of the subretinal fluid (SRF) and cystoid macular edema (CME). **C)** At 24 months, recurrence of SRF and CME were present at the fovea. **D)** SD-OCT shows a hyporeflective space between the choroidal neovascularization (CNV) and Bruch’s membrane (cleft) (white arrows). Recurrent CME and SRF are also seen. Additional photodynamic therapy combined with intravitreal ranibizumab injections was performed. **E**, **F)** At 1 month after additional treatment, the hyporeflective space between the CNV and Bruch’s membrane has completely resolved. CNV beneath the retinal pigment epithelium is attached to Bruch’s membrane.

**Figure 4:**
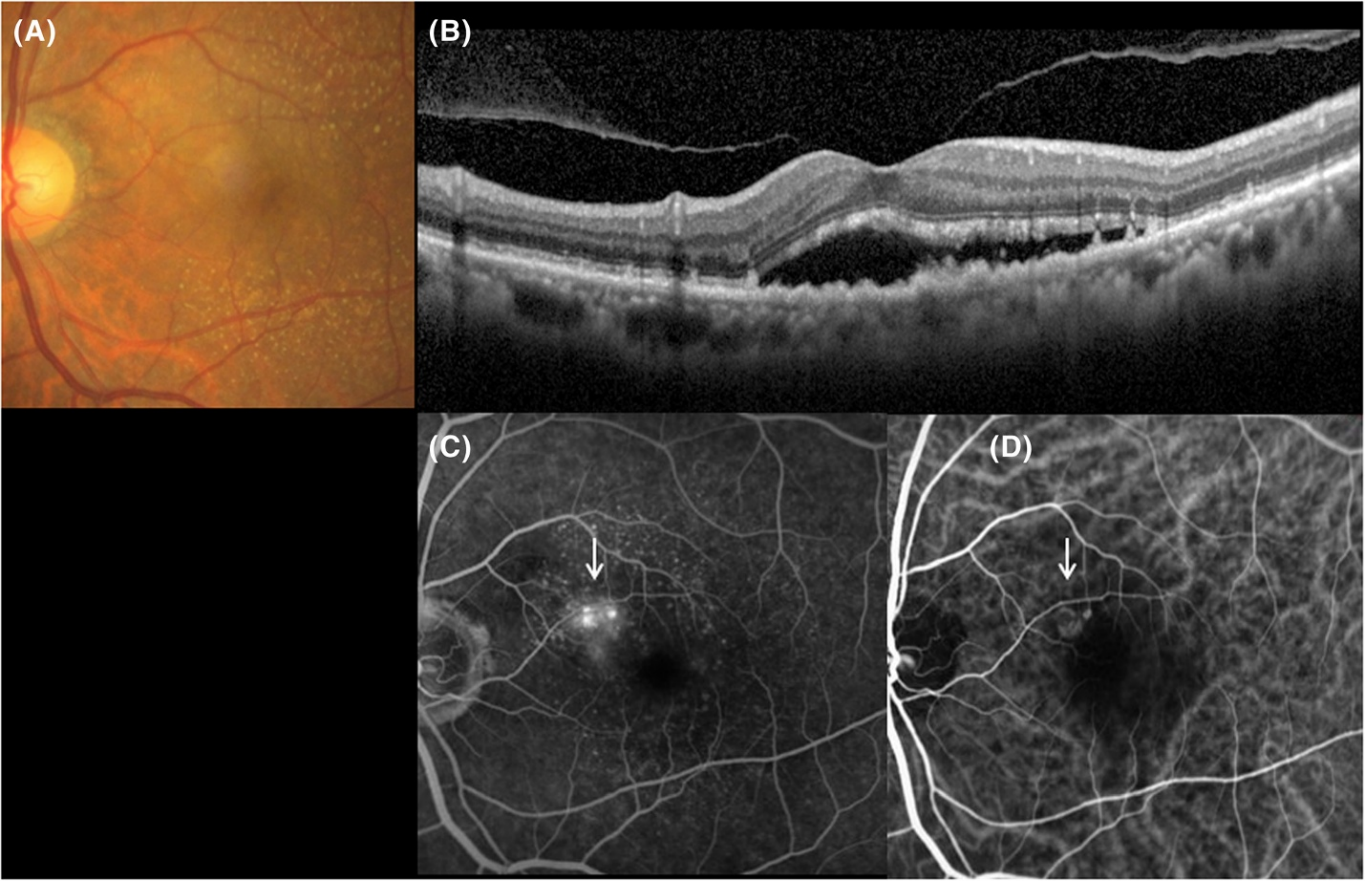
**Initial presentation of Case 2.** A typical occult AMD with no classic choroidal neovascularization (CNV) in the left eye. **A**, **B)** Fundus photography and spectral-domain optical coherence tomography (SD-OCT) show subretinal fluid with pigment epithelial detachment at the fovea. **C)** Fluorescein angiography shows occult CNV (white arrow). **D)** Indocyanine green angiography shows a small abnormal vascular network (white arrow), which was treated with photodynamic therapy combined with intravitreal ranibizumab injection.

**Figure 5:**
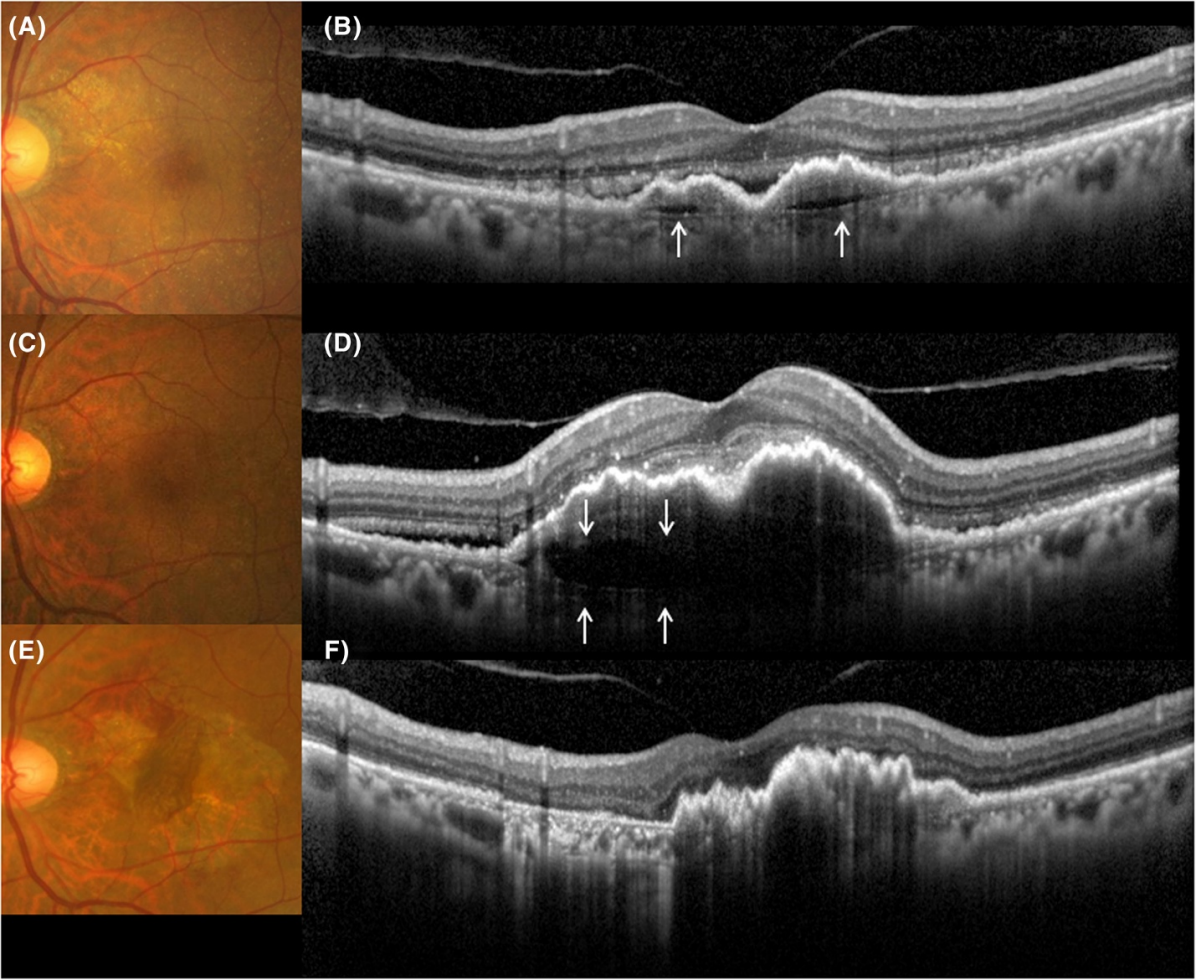
**Clinical course of the cleft in Case 2.** Clinical courses of fundus and spectral-domain optical coherence tomography (SD-OCT) in Case 2 at 3 months **(A, B)**, 6 months **(C, D)** and 9 months **(E, F)** after first treatment. **A)** Subretinal fluid (SRF) was decreased 3 months after first treatment **(B)**. A thin hyporeflective space (white arrows) developed between the sub-retinal pigment epithelium (RPE), choroidal neovascularization (CNV) and Bruch’s membrane (cleft). **C**, **D)** The hyporeflective space thickened gradually with recurrence of SRF at 6 months after first treatment. The left eye was then treated with additional photodynamic therapy combined with intravitreal ranibizumab injection. **E)** At 9 months after first treatment, although the SRF had resolved, RPE tear developed on the temporal side of the macular region. **F)** SD-OCT shows disappearance of the cleft, with the regressed sub-RPE CNV mass with corrugated RPE displaced to the opposite side of the RPE tear.

Clinical characteristics of the patients are shown in Table 1. Of the 227 eyes, 22 (10%; average year: 78.9 ± 6.8, Male: Female = 13: 9) had the cleft. Prevalence of the cleft by lesion subtype was as follows: 12 (18%) of 67 eyes had typical occult AMD with no classic CNV, two (5%) of 38 eyes had typical AMD with predominantly classic CNV, five (5%) of 109 eyes had PCV, and three (23%) of 13 eyes had RAP. A significant correlation was seen between the four types of AMD and emergence of the cleft sign (*p* = 0.007632), while the prevalence of occult AMD with no classic component and RAP tended to be higher than that of the other two types. The superior border of the cleft included the inferior edge of the materials in PED and Bruch’s membrane, which lay along the inferior border of the cleft. The materials in PED were attached to the RPE monolayer along the superior edge of these materials in 18 cases. In two of three eyes with RAP and in two of 12 eyes with occult CNV, these materials were partly detached from the RPE monolayer at the edge of the PED. Mean maximum thickness of the cleft was 61.2 ± 41.0 μm (range, 21-146 μm), while mean maximum thickness of the materials above the cleft was 404.2 ± 256.5 μm (range, 129-1,042 μm). The clinical ramifications of the cleft are summarized in Table 2. At the initial visit to our clinic, 10 of 22 eyes had a cleft, while another 12 eyes developed the cleft during the clinical course (Table 2). The cleft in 14 eyes resolved or decreased with regression of the materials in PED, while the cleft remained unchanged in seven eyes during the entire observation period. In one eye, thickness of the cleft decreased, but did not completely resolve. In 19 (86%) of 22 eyes with a cleft, the patient also showed serous retinal detachment (SRD) or cystoid macular edema (CME). The cleft redeveloped in two of 14 eyes in which it had initially resolved. An RPE tear developed in five (23%) eyes with a cleft. Figures 2, 3, 4 and 5 illustrate representative examples of the cleft and the clinical course before and after treatment.

**Table 1:** **Baseline Features of the Study Patients**

Patinent demographics	
Demographics	
Gender	
Male	163
Female	60
Age(years)	
Mean	74.2 ± 8.7
Range	50-92
The prevalence of the patients with cleft in every lesion subtypes	
All types	22/227 (10%)
Classic	2/38 (5%)
Occult with no classic	12/67 (18%)
Polypoidal choroidal vasculopathy	5/109 (5%)
Retinal angiomatous proliferation	3/13 (23%)

**Table 2:** **Demographic and Clinical Features of Patients with Age-Relate** **d Macul** **ar Degeneration and a Hyporeflective space between hyperreflective materials in pigment epithelial detachment and Bruch’s membrane**

Patient No	Type of AMD	Treatment	Clinical course of the hyporeflective space	Case with disappearance of the hyporeflective space	Case with recurrence of the hyporeflective space	Accompanied with SRD or CME	Accompanied with RPE tear			Follow up period
		PDT with ranibizumab	Case with the hyporeflective space at initial visit					Initial VA	Final VA	
1	Occult		+	-	-	+	-	20/250	20/200	10
2	Occult	PDT with ranibizumab	-	+	-	+	+	20/16	20/250	22
3	Occult	PDT with ranibizumab	-	+	-	+	-	20/32	20/25	22
4	Predominantly classic	PDT with ranibizumab	+	+	-	-	-	20/60	20/50	21
5	Occult	PDT with ranibizumab	-	+	-	-	-	20/60	20/200	22
6	Occult	PDT with ranibizumab	-	+	-	+	+	20/200	20/200	9
7	RAP	PDT with ranibizumab	-	-	-	+	+	20/20	20/100	21
8	Occult	PDT with ranibizumab	+	+	-	+	-	20/60	20/250	18
9	Occult	PDT with ranibizumab	-	-	-	+	-	20/80	20/1000	13
10	RAP	PDT with ranibizumab	-	-	-	+	-	20/80	20/60	18
11	Occult	PDT with ranibizumab	+	+	-	+	-	20/125	20/125	17
12	RAP	PDT with ranibizumab	+	+	+	+	-	20/250	20/125	18
13	Predominantly classic	PDT with ranibizumab	-	-	-	-	-	20/20	20/125	22
14	Occult	PDT with ranibizumab	-	-	-	+	-	20/30	20/40	22
15	Occult	PDT with ranibizumab	-	+	-	+	-	20/50	20/40	16
16	Occult	PDT with ranibizumab		+	-	+	-	20/30	20/100	13
17	Occult	PDT with ranibizumab		+	+	+	-	20/125	20/100	14
18	Occult	PDT with ranibizumab	+	+	-	+	-	20/50	20/40	13
19	PCV	PDT with ranibizumab	-	-	-	+	+	h.m	20/30	7
20	Occult	PDT with ranibizumab	+	-	-	+	-	20/40	20/500	12
21	Occult	PDT with ranibizumab	+	+	-	+	-	20/50	20/250	22
22	Occult	ranibizumab	+	+	-	+	+	20/20	20/60	12

AMD = age related macular degeneration; RAP = retinal angiomatous proliferation; PCV = polypoidal choroidal vasculopathy; PDT = photodynamic therapy; SRD = serous retinal detachment; CME = cystoid macular edema; RPE = retinal pigment epithelium H.M = hand motion.

## Discussion

The current study describes the clinical and conformational characteristics of the cleft, which was located between the materials in PED and Bruch’s membrane in eyes with AMD. We believe that this cleft may represent a space that results from fluid accumulation that originates from active CNV components in the materials in PED. The space between the materials in PED and Bruch’s membrane that we describe here may have the same spatial characteristics originally identified by Khan et al. in cases with old PCV [11] and Nagiel et al. in eyes with RPE tear [12]. Those investigators hypothesized that the PCV lesions, the associated occult CNV, and the remainder of Bruch’s membrane all detached from the underlying choroid, suggesting that the polyps and associated neovascular tissue may be more adherent to the basal surface of the RPE than to the underlying choroid.

Since the cleft resolved or decreased after intravitreal injection of an anti-vascular endothelial growth factor (anti-VEGF) agent in 12 of 22 eyes, the cleft may be filled with exudative fluid due to leakage from the CNV components in the materials. In the current study, 19 (86%) of 22 eyes with a cleft also had SRD or CME. These results may support the possibility that the space between the materials in PED and Bruch’s membrane comprises fluid from these materials that may include CNV membrane. In cases with a cleft, leakage from CNV may flow anteriorly toward the retina and posteriorly toward Bruch’s membrane. In 10 (45%) of 22 eyes with a cleft, we observed the clefts before treatment with anti-VEGF agents or PDT combined with anti-VEGF therapy was initiated, with a cleft developing after these treatments in 12 (55%) of 22 eyes. The mechanism by which clefts develop may thus have two distinct contributory factors. First, leakage from the materials that may include CNV membrane posteriorly toward the choroid may be an important factor. Second, contraction of the materials, which occurs after treatment with anti-VEGF drugs or PDT, or spontaneously, may also represent a key step. The cleft in Cases 12 and 17 resolved after the anti-VEGF treatment, but redeveloped over time in both of these cases. The composition of materials in PED were speculated and the components could represent one or any combination of three materials: 1) proteinaceous exudate; 2) fibrocellular proliferation; or 3) fibrovascular proliferation [3]. The materials in PED in our study could also have had the same composition, and these components may show leakage in some cases.

Because contraction of the materials in PED after treatment with an anti-VEGF drug or PDT along with increased tension in the RPE monolayer resulting in detachment of the vascularized RPE due to leakage from the CNV may be crucial steps in the development of RPE tears, cases with AMD in which a cleft emerges may need to be closely observed for the development of RPE tear. A space between the materials beneath RPE and Bruch’s membrane may enable the materials to contract independently of Bruch’s membrane. Of the 22 eyes with a cleft, RPE tear developed in five eyes (23%) after treatment. Previous studies have reported that RPE tears developed in eyes with a highly vascularized PED [15–18]. In fact, of the five eyes with RPE tear in the current study, all had a large vascularized PED [3].

These results suggest that we may ascertain whether subretinal exudate or RPE tear may follow patients with clefts during the overall clinical course.

A histopathological study by Bressler et al. reported finding an occult AMD lesion and a cleft between the CNV and Bruch’s membrane in some specimens [19]. In these specimens, the RPE monolayer and the CNV beneath the RPE basement membrane were shown to be tightly adhered. However, a basal laminar deposit was found to have intervened between the RPE monolayer and CNV.

Tsujikawa et al. speculated that when the polypoidal lesions in PCV have increased exudates, the fluid from these lesions infiltrates the area under the polypoidal lesions, resulting in detachment of the polypoidal lesions from Bruch’s membrane and giving the appearance of being inside the PED [20]. They reported that while the fluid was beneath the RPE and CNV in PCV, the polypoidal lesions beneath the sub-RPE were at the notch of the PED. However, the lesions under the RPE layer in the present study continued uninterrupted from one side to the other of the PED. While the location of the cleft was the same as the space reported by Tsujikawa, the continuity of the lesions beneath the RPE differed between these two studies.

In this study, clefts were detected in two eyes with predominantly classic CNV. However, OCT showed that both eyes had occult lesions. These clefts may thus have resulted from underlying sub-RPE CNV.

The limitations of the current study were the retrospective nature of the investigation and the small number of eyes with clefts that were examined. Further research on the detection of clefts in a study with a larger number of patients will need to be undertaken in the future.

## Conclusions

A hyporeflective space between hyperreflective materials in PED and Bruch’s membrane can sometimes be observed in patients with neovascular AMD using OCT. The clinical characteristics of the cleft indicate that the materials beyond the cleft may show residual activity.
